# Effect of nutrition counseling on nutritional status and gestational weight gain of pregnant adolescents in West Arsi, Central Ethiopia: a cluster randomized controlled trial

**DOI:** 10.1038/s41598-024-55709-y

**Published:** 2024-03-01

**Authors:** Adane Tesfaye, Dessalegn Tamiru, Tefera Belachew

**Affiliations:** 1 Department of Nutrition and Dietetics, Faculty of Public Health, Institute of Health, Jimma University, Jimma, Ethiopia; 2 Department of Nutrition, School of Public Health, College of Medicine and Health Science, Dilla University, Dilla, Ethiopia

**Keywords:** Pregnant adolescent, Alliance for development, Nutritional Behavior Change Communication, Nutritional status, Health care, Medical research

## Abstract

When pregnancy occurs in adolescence, the growth and development of the mother and fetus may be impaired due to strong competition for nutrients between the still-growing adolescent and the fetus. Pregnant adolescents constitute an underserved population; they lack adequate nutritional knowledge. Therefore, this study investigated the effect of nutritional behavior change communication (NBCC) through alliance for development (AFD) on the nutritional status and gestational weight gain (GWG) of pregnant adolescents. A two-arm parallel cluster randomized controlled community trial was conducted in the West Arsi Zone, central Ethiopia, from August 2022 to July 2023. The nutritional status of the pregnant adolescent was assessed using mid-upper arm circumference. Weight was measured at baseline and at the end of the intervention. A total of 207 and 219 pregnant adolescents participated in the intervention and control clusters, respectively. The intervention started before 16 weeks of gestation, and the intervention group attended four NBCC sessions. The NBCC was based on the health belief model (HBM) and was given at the participants’ homes with their husbands. The NBCC intervention was delivered by AFDs and community-level health actors. Pregnant adolescents in the control group received routine nutrition education from the health care system. A linear mixed-effects model and difference in difference (DID) were used to measure the intervention effect after adjusting for potential confounders. After the implementation of the trial, the mean mid-upper arm circumference (MUAC) in the intervention arm significantly increased from baseline (p ≤ 0.001), 23.19 ± 2.1 to 25.06 ± 2.9 among intervention group and 23.49 ± 2.1 to 23.56 ± 2.0 among control group and the mean difference in the MUAC (DID) was 1.89 ± 2 cm (p ≤ 0.001); the mean GWG in the intervention arm significantly increased from baseline; 51.54 ± 4.7 to 60.98 ± 4.6 among intervention group and 52.86 ± 5.27 to 58 ± 5.3 among control group; the mean GWG in the intervention group was 9.4 kg, and that in the control group was 5.14 kg, and the difference in difference was 4.23 kg and this was statically significant p ≤ 0.001). This study demonstrated that the use of the HBM for NBCC delivered through the AFD was effective at improving the nutritional status and GWG of pregnant adolescents. These results imply the need for the design of model-based nutritional counseling guidelines.

Clinical trial registration: PACTR202203696996305, Pan African Clinical Trials Registry, date of first registration: 16/03/2022.

## Introduction

When pregnancy occurs in adolescence, the growth and development of the mother and fetus may be hampered due to strong competition for nutrients between the still-growing adolescent mother and her fast-developing fetus, commonly known as “nutrient partitioning”^1,2^. Alternative theories that may be used in conjunction with nutrient partitioning include the idea that due to gynecological immaturity, safe delivery is sacrificed to allow for optimal fetal growth^3,4^.

A desirable amount of weight to be gained is associated with optimal outcomes for mothers and infants. A Joint FAO/WHO/UNU Expert Consultation report recommended that healthy, well-nourished women should gain 10–14 kg during pregnancy. This will increase the likelihood of giving birth to full-term infants with an average birth weight of 3.3 kg and to lower the risk of fetal and maternal complications^5,6^.

Behavior change communication (BCC) refers to the methodical application of communication that has a positive impact on people’s understanding, attitudes, and behaviors. This encourages people to take charge of their own health. BCC can promote dietary improvement and account for individual health beliefs and practices^7^. Nutrition behavior change communication (NBCC) is an approach for changing nutrition-related behaviors through the use of different strategies, techniques, and teaching materials to bring about practice changes that lead to improved health through optimal feeding practices and improved dietary habits^8^. BCC is a straightforward strategy for altering behavior. It differs from traditional information education communication (IEC) because, in contrast to BCC, IEC considers only spreading awareness and providing information^9^.

In low- and middle-income (LMIC) countries, as of 2019, there were approximately 21 million pregnancies among teenagers aged 15–19^10^. Regarding the magnitude of child marriage in Eastern and Southern Africa, Ethiopia is among the top three nations. The vast majority of people give birth during adolescence after marrying as children^11–13^. The intergenerational effect of malnutrition has been recognized as a major contributing factor to Ethiopia’s inclusion among countries with the highest rates of maternal and newborn mortality in the world^14,15^.

Pregnancy involves a variety of adverse reactions, including nausea, vomiting, fatigue, heartburn, constipation, hemorrhoids, food cravings and aversions^16^. These problems may be alleviated by the NBCC. Many expectant mothers in developing nations restrict their food intake for different reasons, such as through the false belief that smaller babies will have fewer delivery complications and through the belief that cultural influences will increase the size and difficulty of delivery^17,18^. This illustrates the lack of adequate information and false beliefs about dietary behaviors among expectant mothers^19–21^.

AFDs (alliances for development), originally known as women development armies [WDAs], are community health volunteers who are in charge of 30 houses, each of which has one to five networks. Six different one-to-five connections formed one WDA^22^. Ethiopia is an excellent example of a community health initiative with a well-structured health extension package. The country's primary healthcare unit (PHCU) is at the forefront of PHC^23^. A study conducted on the contribution of the Women’s Development Army or by its new name, AFDs, to maternal and child health showed that they have contributed to the improvement of child immunization service use, the minimization of maternal mortality, improved skilled delivery attendance and skilled ANC^24^, an experience that can be extended to nutrition education, and other developing countries might adapt it.

A systematic review and meta-analysis conducted in 2021 showed that the pooled estimated prevalence of teenage pregnancy in Ethiopia was 23.59%^25^. There are several efforts from the government of Ethiopia to address adolescent health and nutritional problems. For example, adolescent and youth health (AYH) programmes, including those focused on sexual and reproductive health (SRH) and youth development, have gained attention^26^ The National Adolescent and Youth Health (AYRH) strategy of 2007^27^, Comprehensive National Adolescent and Youth Health (AYH) strategy of 2016^28^ and National Nutrition Programme (NNP II) 2016–2020^29^; however, these programmes were not effective at the expected level because adolescents and youth-related interventions in Ethiopia are uncoordinated, fragmented under various ministries, lacked effective policy implementation, underfunded, project oriented, and lacked meaningful engagement from young people^30^.

Compared to adult women, pregnant at any age during adolescence is associated with a greater possibility of experiencing eclampsia, puerperal endometritis anemia, systemic infections, low birthweight, preterm delivery, and neonatal mortality^31–33^. Pregnant adolescents constitute an underserved population; therefore, they can benefit greatly from receiving prenatal health and nutritional education. Adolescents often experience unplanned pregnancy. Several studies have shown that as few as 27% of pregnant adolescents receive ANC services^34,35^, which makes them even more vulnerable to undernutrition and other adverse health outcomes. To the best of our literature search, no community-based interventional study has investigated the effect of a behavioral change model-based NBCC through community-level health volunteers (AFDs) on nutritional status and weight gain among pregnant adolescents; therefore, this study can help policymakers and planners improve nutritional counseling practices at local and national levels.

## Methods

### Study design, setting and participants

This study was a cluster randomized controlled community trial with a two-arm parallel design that lasted for one year. This study was conducted in the West Arsi Zone, Oromia region, Central Ethiopia; 250 km from Addis Ababa, the nation's capital. There are 16 districts in the West Arsi Zone (13 rural districts and three towns). A total of 2,929,894 people were estimated to live there by mid-2022. A total of 12,556 km^2^ make up the zone, which has a climate of 45.5% highland, 39.6% medium land, and 14.9% lowland^36^. According to the Zonal report of 2022, there are 417 public health facilities, 5 of which are hospitals, 324 of which are health posts, 88 of which are health centers, and 203 of which are private medium and higher clinics, including one nongovernmental hospital and two private hospitals providing health services. At least one sexual and reproductive health (SRH) service was utilized in the zone; in 2019, it was 58.6%^37^. The study was conducted between August 2022 and July 2023. This trial included pregnant adolescents before 16 weeks of pregnancy who intended to remain in the study region until delivery. Adolescents who refused to give their informed consent were not included in the study.

### Sample size determination and sampling technique

The sample size was calculated using G*Power 3.0.10. The required sample size was determined using the following assumptions: a 95% confidence interval, a 5% margin of error, 80% power, and an intra-cluster correlation (ICC) of 0.03 (based on a comparable published study ICC)^38^ for the difference between two independent means (two groups). A 10% loss to follow-up was considered, and a design effect of two was employed. A total of 488 people constituted the computed sample sizes. As a result, both intervention and control groups of 244 pregnant adolescents were included. Twenty-eight clusters were used, and the average cluster size was 19. A single-stage cluster sampling technique was applied in this study.

### Recruitment, randomization and intervention allocation

**Figure 1:**
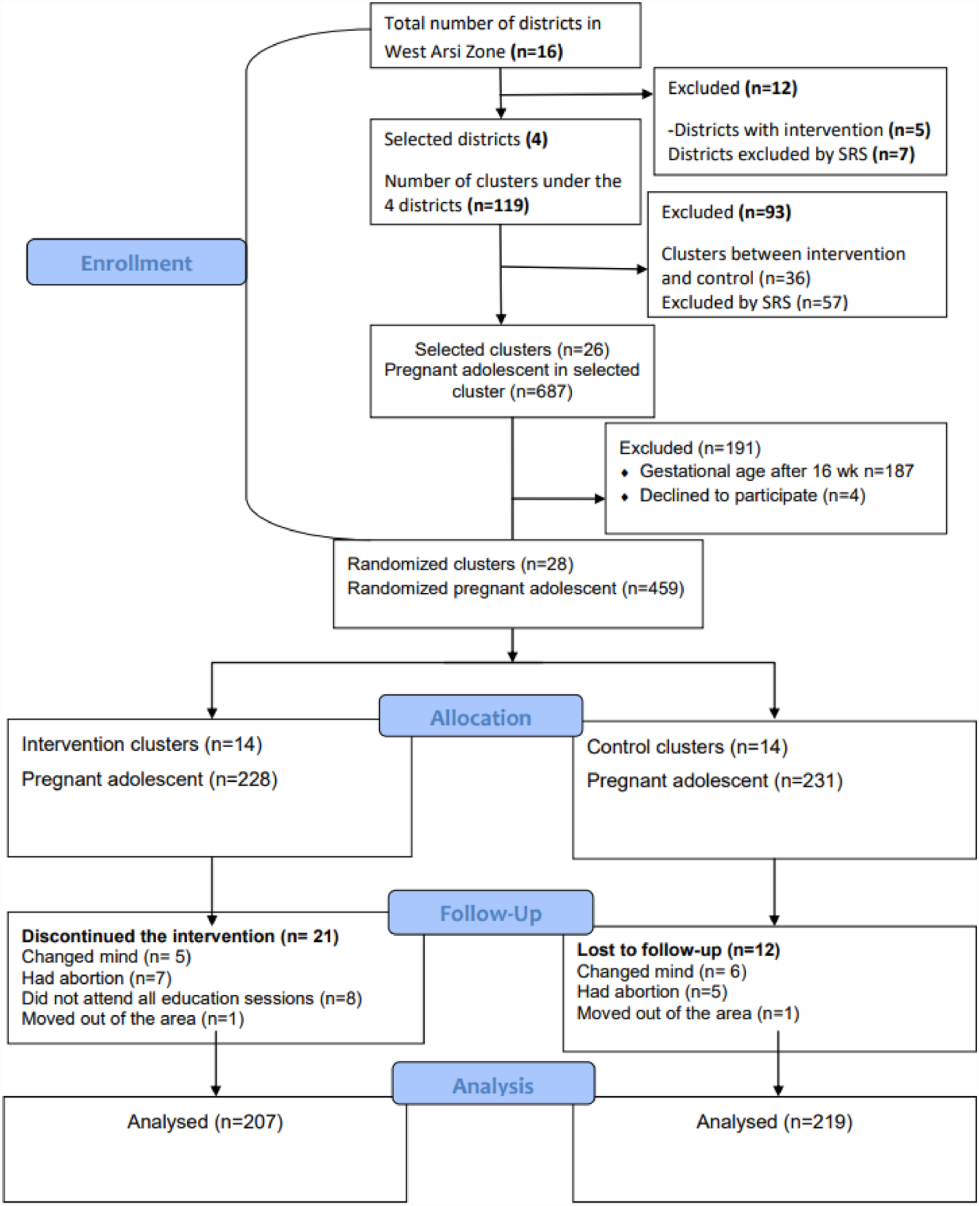
Flow of the study participants through the trial according to the criteria recommended in the CONSORT guideline.

Five of the zone's 16 districts had nutritional education interventions; thus, they were excluded from the study. Four districts, namely, Dodola Rural, Adaba, Gedeb Hasasa, and Siraro, were chosen from among the remaining eligible districts by simple random sample (SRS) technique. Samples of nonadjacent kebeles (clusters) from the four districts were chosen using SRS. Six clusters each from the Dodola Rural and Adaba districts, as well as eight clusters each from the Gedeb Hasasa and Siraro districts, were selected based on their proportion to size allocation and considering cluster allocation in the intervention and control groups. Kebeles (the smallest administrative entity in Ethiopia) were used as the randomization unit (clusters). Clusters for the intervention and control groups were distributed using the lottery (SRS) approach. The Consolidated Standards of Reporting Trials (CONSORT) guidelines were used to report the results (Fig. 1).

A cluster randomized trial was used to avoid message contamination because pregnant teenagers in the same cluster were likely to communicate and discuss intervention messages. To avoid information leakage, all pregnant adolescents who met the criteria in one cluster were enrolled in the same arm (either the intervention or control arm). Buffer zones (none selected clusters) were also positioned^39,40^.

The study included all pregnant teenagers who met the inclusion criteria. The study participants were screened and enrolled by nurses, and the clusters were randomly assigned. A house-to-house survey was performed, and pregnant teenagers who met the criteria were screened by first date of their last menstrual period and urine HCG test was used to confirm their pregnancy. Urine-based pregnancy tests and urine HCG levels were used. The procedure involved dipping a test strip into a urine sample. The test strip contained chemicals, monoclonal antibodies that reacted to the presence of the pregnancy hormone HCG (human chorionic gonadotropin). The results are displayed as lines on the test strip.

### Intervention

The intervention strategy used in this study was a community-based nutritional behavioral change communication intervention (NBCC) based on the Health Belief Model (HBM). The recommendations of the World Health Organization, the blended training module on nutritional counseling developed by the Federal Ministry of Health of Ethiopia, EFDRE/MOH, and related interventional studies^41–44^ served as the basis for preparing the intervention package. Additionally, the baseline study, conducted at the start of this study, served as a direction for the development of intervention tools; the NBCC included the husbands of the pregnant teens and a demonstration of how to prepare meals. The intervention items included a training manual for nutrition counsellors, leaflets with key messages for pregnant adolescents and their families, and counseling checklist cards.

The intervention method was tested for one week in an environment similar to the research site, and adjustments were made in light of the results. The counseling manual's core contents included eating a range of foods, especially iron-rich foods, animal products, fruits, and vegetables, and increasing the meal frequency and portion size as gestational age increased. The main components of counseling guidance also included taking iodized salt and iron/folic acid supplements. Additional messages about the core components included reduced workload, day rest, the use of impregnated bed nets, and the use of medical services.

It was also emphasized how undernutrition can harm a person’s development and how vulnerable pregnant adolescents need to eat. The benefits of eating enough meals that are diverse and the challenges in maintaining a balanced diet were also highlighted in the NBCC guidebook. Throughout her pregnancy, each pregnant adolescent attended four counseling sessions. Personalized NBCC based on trimester was provided during home visits. Counselors used a client-centered approach to identify specific dietary preferences and needs. Before allowing the teenagers to choose easily accessible, agreeable, and affordable guidance at their location, counselors considered the needs of the pregnant adolescents, their household income, and any gaps they had discovered. Counseling was conducted using the GALIDRAA approach (Greet, Ask, Listen, Identify, Discuss, Repeat, Agree, and Appoint)^45^.

A counseling guide that included the necessary information was used to conduct each counseling session for the NBCC, which lasted 45–60 min. The first appointment concentrated on the basics of nutrition, food groups, selecting foods that provide a balanced diet, showing how to prepare meals, the frequency of meals, and the use of iodized salt before 16 weeks of pregnancy. The second and third counseling sessions, which covered the whole of the counseling manual, were offered during the second trimester. Final counseling, which focuses on weight gain and incorporates all the module's important messages, was provided based on the gaps that were discovered during the previous trimesters of pregnancy.

Each pregnant adolescent in the intervention arm received a leaflet containing the key themes in Afan Oromo and Amharic (local languages) and appropriate images. Anyone at home who could read was asked to read the leaflet to the pregnant adolescent if the adolescent could not read it.

Health extension workers chose 14 AFD [formerly known as WDAs] counselors based on their performance and prior involvement with public health services. The 14 AFDs were carefully supervised by four BSc nurses. Role-playing exercises and fieldwork using the training handbook were part of rigorous one-week training for counselors and supervisors. After the intervention had been in place for 2 months, the supervisors and counsellors received three additional days of training to ensure that the providers continued to adhere to standardized practices.

### Intervention fidelity

Criteria were created to assess the integrity of the intervention based on the best practice suggestions developed by the National Institutes of Health Behavioral Change Consortium^46^. The criteria^47^, included checklists to assess the intervention design, counselor training, counseling process, receipt of the intervention, and implementation of the skills picked up during the intervention. Nonadjacent clusters were selected to prevent information contamination. The intervention and control groups had an equal number of clusters from each district to balance differences.

The intervention strategy was tested before the experiment. Additionally, each pregnant adolescent received the same number and frequency of counseling sessions, and the lengths of interactions within the intervention group were comparable to standardize the method. Counselor training was given in a group environment using a training booklet, role-playing, and simulated counseling sessions. Tests administered before and after training, as well as a practical evaluation, were utilized to evaluate counselors’ skills and knowledge. The process observer graded the counselors using a “yes/no” rating system and looked at things such as using a counseling guide, covering the whole subject, the duration and frequency of counseling, preparation, accuracy, and the counselor’s ability to respond to questions appropriately. Pregnant adolescents’ understanding of food throughout pregnancy was assessed through interviews via checklists to determine their knowledge of the main components of the intervention.

Participants, counselors, and data collectors were blinded to the study’s objective; participant allocation concealment was impractical given the nature of the intervention. Until the analysis was complete, the groups were given a unique nonidentifiable number that also served to blind the data entry clerk. The counseling process was supervised by the main investigator and counseling supervisors.

### Data collection procedure and measurements

The primary outcome of this study was nutritional status as measured by the MUAC, while the secondary outcome was GWG. The mid-upper arm circumference (MUAC) is the recommended assessment tool for nutritional status because of its simplicity and sensitivity in detecting undernutrition. In low-resource settings, where girls have minimal subcutaneous fat, it is the preferred measurement method because changes in the MUAC are more likely to reflect changes in muscle mass^48^. It has been demonstrated that a low maternal MUAC is useful for detecting unfavorable delivery outcomes, such as intrauterine growth restriction, preterm birth, and asphyxia at birth^49^. The left mid-upper arm circumference (MUAC) was measured at the anatomical landmark at the midpoint of the acromion and olecranon processes of the nondominant hand, with the palm facing upward and the women's elbows flexed to 90°. The measurements were taken twice by employing inelastic MUAC tape and interpreting the measurements to the nearest 0.1 cm.

A pretested structured questionnaire was used to obtain the data. Sociodemographic characteristics, meal frequency, use of medical services, types of diet, and Household Food Insecurity Access Scale (HFIAS) scores were collected. Six clinical nurses and two master of public health (MPH) holders worked as data collectors and supervisors, respectively. Pregnancy tests were performed by three female laboratory technologists. The data collectors conducted in-person interviews with the participants at their homes to administer the questionnaire. To the best extent possible, the adolescent's privacy was protected by prohibiting access to the site where the interviews took place.

According to the Food and Nutrition Technical Assistance (FANTA) III recommendation from the United States Agency for International Development (USAID) and the Food and Agricultural Organization (FAO), dietary intake was determined using 24-h recalls^50^. The objective was to ascertain whether the meals consumed by pregnant teenagers varied. Ten food groups were used to compute dietary diversity; grain, dairy, meat, white roots and tubers; nuts and seeds; eggs; dark green leafy vegetables; poultry and fish; plantains; other fruits and other vegetables; and other fruits and vegetables high in vitamin A are among the ten food groups listed in the recommendations. Adequate dietary diversity is achieved if pregnant adolescents consumed food from 5 or more food groups within 24 h of day/before data collection; minimum dietary diversity for women (MDD-W) was used^51,52^. The participants were asked to remember every meal they consumed during the previous 24 h; both inside and outside the home. The participants were also asked if they could remember any between-meal snacks they may have consumed. Food items received a “1” rating if consumed during the reference period and a “0” rating if not.

The HFIAS (Household Food Insecurity Access Scale) Guideline^53^ was used to assess food security. The HFIAS was used to evaluate the households’ level of food security and included nine questions. Prior to this, the questions were validated for use in developing nations^54^. Food-secure households experienced fewer food insecurity indicators than did the first two food insecurity indicators. Households that experienced between two and ten, eleven to seventeen, or more than seventeen food insecurity indicators were considered mildly, moderately, or severely food insecure, respectively.

The household’s wealth index was derived using principal component analysis (PCA), taking into account access to a latrine, a water source, household durable assets, and agricultural land. The responses to the none dummy variables were all split into three groups. A code of 1 is allocated to the highest rating. The two smaller digits, however, were given a code of 0. Using variables with a commonality value larger than 0.5, PCA factor scores were produced. The first primary component score for each family is kept to calculate the wealth score. Quintiles of the wealth score were created to classify households as poorest, poor, medium, rich, or richest^55^.

The autonomy of the pregnant adolescents was evaluated using eight questions. Code one was provided for each question when a decision was made by the girl, by herself or jointly with her husband; otherwise, code zero was provided. The mean score was used to determine the pregnant adolescent's decision-making capacity^44^.

### Data management and analysis

The data were entered using the Kobo Toolbox and exported to SPSS version 25 for analysis. The baseline variations in the two groups’ sociodemographic characteristics were examined using a Chi-square test. Paired *t* tests and independent *t* tests were used to determine within- and between-group differences.

The effect of the intervention on changes in the nutritional status and gestational weight gain of pregnant adolescents over time was estimated using a linear mixed-effects model. This model was chosen because of the repeated assessments (pre- and postintervention) and the clustering of individuals, which allowed us to explain how the results were correlated. The Akaike information criterion (AIC) was used to help us choose the best statistical model. The model that displayed the lowest AIC was selected. The bivariate linear mixed regression model's variables with p 0.2 were chosen as potential candidates for the multivariate linear mixed model analysis. By analyzing how time and the intervention interacted, the effectiveness of the intervention was evaluated.

Participants were examined as random effects during model fitting. A linear mixed-effects model also makes it possible to manage the impact of various confounding variables. The intra-cluster correlation coefficient of the final model was 0.04, indicating that constructing a third-level model was not necessary. The intercept-only model was created initially. To take into consideration time-invariant variables at the individual level, a two-level model was fitted. The effect of intervention was estimated by testing the interaction term between treatment allocation and time.

### Ethical considerations

Ethical approval was obtained from the Jimma University IRB/ethics committees with reference number JUIH/IRB/194/22, and the Oromia Regional Health Office provided support. All methods were performed in accordance with the relevant guidelines and regulations; the study was performed in accordance with the Declaration of Helsinki. Each study participant received a thorough description of the study's title, goal, protocol, and duration, as well as the potential risks and benefits, prior to providing informed consent. Each teenager provided verbal, written, and signed informed consent prior to any interview or measurement. Informed consent was obtained from the LAR (legally authorized representative) for study participants aged 18 years and younger. Participants were made aware of the publication of their anonymous comments. Informed consent was obtained from participants prior to the commencement of interviews. The researcher remained truthful to the academic and ethical requirements. Finally, the researcher kept the data in a locked file cabinet in a safe place after the completion of the study. Informed consent was obtained from both the adolescent and their husband or parents. Finally, any ethical issues that arose during this research were resolved through discussion between the researcher and JU’s IRB.

## Results

The overall follow-up of the study participants throughout the trial was summarized in the CONSORT guideline flow chart (Fig. 1).

### Sociodemographic characteristics of pregnant adolescents

Of the 459 participants initially sampled, 426 (IG = 207, CG = 219) pregnant teenage participants in this study followed the protocol exactly and were included in the analysis. The mean follow-up week was 15 weeks. The sociodemographic features of the intervention and control groups did not significantly differ at baseline according to the Chi-square test (p > 0.05). The baseline characteristics of the pregnant adolescents are shown in Table 1.

**Table 1:** Sociodemographic characteristics of pregnant adolescents in the West Arsi Zone, central Ethiopia, 2022–2023.

Variables	Intervention group (n1 = 207) Frequency %	Control group (n1 = 219) Frequency %	p value
Number of clusters	14	14	
Age			
Middle adolescent	51 (24.7)	46 (21)	0.37
Late adolescent	156 (75.3)	173 (79)	
Family size			
< 5 persons	148 (71.5)	173 (79)	0.07
> = 5 person	59 (28.5)	46 (21)	
Marital status			
Married	174 (84)	188 (85.8)	0.60
Unmarried/divorced	33 (16)	31 (14.2)	
Educational status			
No formal education	20 (9.6)	30 (13.6)	0.20
Can read and write	27 (13)	26 (11.8)	
Primary level education	113 (54.5)	102 (46.5)	
Secondary education	35 (16.9)	52 (23.7)	
College and above	12 (5.7)	9 (4)	
Occupation			
House wife	71 (34.3)	64 (29.2)	0.9
Student	77 (37)	86 (39.3)	
Merchant	34 (16.4)	39 (17.8)	
Daily laborer	7 (3.4)	9 (41)	
Farmer	9 (4.3)	9 (41)	
Government job	9 (4.3)	12 (5.4)	
Wealth index			
Poorest	42 (20)	46 (21)	0.67
Poor	32 (15.4)	48 (21.9)	
Medium	32 (15.4)	17 (7.7)	
Rich	59 (28.5)	70 (32)	
Richest	42 (20.3)	38 (17.3)	

### Health belief model construct scores and their correlation with the MUAC and GWG

At baseline, there was no significant difference in the HBM construct scores between the intervention and control groups. All the HBM constructs had a significant positive correlation with the nutritional status and GWG of the pregnant women, except for the perceived barrier (p < 0.05) (Table 2).

**Table 2:** Correlations of the health belief model constructs with MUAC and GWG among pregnant adolescents in West Arsi, central Ethiopia, 2022–2023.

	Perceived susceptibility	Perceived severity	Perceived benefits	Perceived barriers	MUAC	GWG
Perceived susceptibility	1					
Perceived severity	0.943**	1				
Perceived benefits	0.917**	0.869**	1			
Perceived barriers	0.09	0.107	0.164	1		
MUAC	0.145**	0.150**	0.191**	− 0.211	1	
GWG	0.173**	0.181**	0.194**	0.068	0.190**	1

*GWG* gestational weight gain, *MUAC* mid-upper arm circumference.

*Correlation is significant at the 0.05 level (2-tailed), and **correlation is significant at the 0.01 level (2-tailed).

### Effect of nutritional behavioral change communication on the nutritional status of pregnant adolescents

At baseline, there was no statistically significant difference in the mean MUAC (23.19 ± 2.1 vs 23.49 ± 2.1, p = 0.20) between the two groups. After the implementation of the trial, the mean MUAC in the intervention arm significantly increased from the baseline (p = < 0.001) (Table 3).

**Table 3:** Differences between baseline and end line MUAC and gestational weight gain and differences in the DID between the intervention and control groups among pregnant adolescents in West Arsi, central, Ethiopia, 2022- 2023.

Variable	Study period	Intervention group Mean (± SD)	Comparison group Mean (± SD)	DID Mean (± SD)
MUAC	Baseline	23.19 ± 2.1	23.49 ± 2.1	1.89 ± 2***
	End-line	25.06 ± 2.9	23.56 ± 2.0	
	Difference (EL-BL)	1.87 ± 1.67	0.7 ± 0.4	
Gestational weight gain	Baseline	51.54 ± 4.7	52.86 ± 5.27	4.23 ± 0.34**
	End-line	60.98 ± 4.6	58 ± 5.3	
	Difference (EL-BL)	9.44 ± 4.48	5.14 ± 3.7	

*BL* baseline, *EL* end-line, *SD* standard deviation, *CI* confidence interval, *MUAC* mid‐upper arm circumference; **p < 0.01; ***p < 0.001.

The variance in the individual-level residual errors or variability in the average MUAC across individuals was 2.79, which was statistically significant (p = < 0.001). The intraindividual correlation coefficient was 0.55, which indicated the importance of accounting for individual-level time-invariant variables (fitting a two-level model). After controlling for food security, wealth index, DDS, and meal frequency, the intervention group showed a significant improvement in nutritional status at the end of the study (p < 0.01) (Table 4).

**Table 4:** Linear mixed‐effects model predicting the MUAC of pregnant adolescents in the West Arsi Zone, Central Ethiopia, 2022/2023.

Fixed effect	Model 1		Model 2		Model 3	
	Estimate (SE)	95% CI	Estimate (SE)	95% CI	Estimate (SE)	95% CI
Intercepts	23.78 (0.1)	23.5–23.9	24.7 (0.33)	24–25.37	23.7 (0.7)	22.3, 25.13
Intervention effect			0.65 (0.2)	0.27, 0.83	0.65 (0.2)	0.27, 0.83
Baseline MUAC			− 0.253 (0.3)	− 0.4, − 0.14	− 0.253 (0.3)	− 0.4, − 0.14
End-line MUAC			− 0.72 (0.27)	− 0.92, − 0.47	− 0.36 (0.27)	− 0.53, 0.28
DDS					0.6 (0.29)	0.06, 1.2
Wealth index					− 0.01 (0.06)	− 0.14, 0.12
Food security					0.13 (0.2)	0.08–0.27
Meal frequency					0.08 (0.24)	− 0.4, 0.13
Random effect						
Level 2 variance	2.79 (0.3)		2.78 (0.25)		2.26 (0.18)	
ICC	0.55		0.53		0.04	
AIC	3796		3791.9		3796	

*AIC* Akaike information criterion, *DDS* dietary diversity score, *ICC* intra-cluster correlation, *SE* standard error, *CI* confidence interval.

### Effect of nutritional behavioral change communication on gestational weight gain (GWG) in pregnant adolescents

At baseline, there was no statistically significant difference in the mean GWG in kg (52.86 ± 5.27 vs 51.54 ± 4.7, p = 0.34) between the two groups. After the implementation of the trial, the mean GWG in the intervention arm significantly increased from baseline. The mean weight gain was 9.4 kg in the intervention group and 5.14 kg in the control group; the difference was 4.23 kg, and this difference was statistically significant (p = < 0.001) (Table 3).

The variance of the individual-level residual errors of the average GWG across individuals was 5.53, which was statistically significant (p = < 0.001). The intraindividual correlation coefficient was 0.139, which indicated the importance of accounting for individual-level variables (fitting a two-level model). After controlling for age, occupation, DDS, female decision-making and meal frequency, the intervention group showed significant improvement in GWG at the end of the study (β = 5/59, p < 0.01) (Table 5).

**Table 5:** Linear mixed‐effects model predicting gestational weight gain in pregnant adolescents in the West Arsi Zone, Central Ethiopia, 2022–2023.

Fixed effect	Model 1		Model 2		Model 3	
	Estimate (SE)	95% CI	Estimate (SE)	95% CI	Estimate (SE)	95% CI
Intercepts	55.85 (0.23)	55.4–56.3	55.9 (0.23)	55.46–56.4	55.97 (1.6)	52.8, 59.2
Intervention effect			5.59 (0.7)	4.1, 6.98	5.59 (0.7)	4.1, 6.98
Female decision making					− 0.85 (0.57)	− 1.99, 0.28
DDS					2.42 (0.63)	1.17, 3.67
Age					− 0.12 (0.4)	− 1, 0.8
Occupation					0.028 (0.16)	− 0.28, 0.34
Meal frequency					0.55 (0.5)	− 0.53, 1.64
Random effect						
Level 2 variance	5.53 (1.89)		3.26 (1.57)		3.04 (0.83)	
ICC	0.139		0.066		0.03	
AIC	5546		5357.5		4838.5	

*AIC* Akaike information criterion, *CI* confidence interval, *ICC* intra-cluster correlation, *SE* standard error.

## Discussion

This study showed that the nutritional status and GWG of pregnant adolescents were improved by NBCC delivered through AFDs using the HBM. The mean MUAC and GWG of adolescents in the intervention group were significantly greater than those of adolescents in the comparison group. These results persist after controlling for potential confounders.

The finding on improvement of nutritional status is consistent with those of Eastern Shoa Zone, Ethiopia^44^; West Gojjam Zone, Ethiopia^43^; Lahore, Pakistan^56^, and Iran^57^, who reported significant improvements in the nutritional status of pregnant individuals who attended nutritional education. A possible explanation for these findings is that human behavior can be changed by well-designed adequate nutritional education, which ultimately leads to improvements in nutritional status and better health.

Another important finding is that the NBCC resulted in significant improvement in the GWG of pregnant adolescents within the recommended weight gain range, and these results are consistent with other research^58–60^. This might be because the NBCC made participants change their perception and increase their understanding of good nutrition.

For the HBM constructs, perceived barriers were the main problem among participants, and perceived benefits brought about more significant changes. This finding is consistent with the findings of other studies^61–63^. Perceived barriers are often the most predictive constructs and are often more difficult to change than other constructs; therefore, more attention needs to be given to perceived barriers. Addressing perceived barriers often requires considering and addressing the broader context in which the behavior occurs. Perceived barriers are influenced by various contextual factors, such as social, cultural, economic, and environmental factors^64,65^.

The findings from this study provide some evidence that carefully selected, intensively trained, and closely supervised AFDs can be used actively in the health care system for NBCC and other similar health care services, thereby decreasing the overload on health care providers. These are promising findings, especially for developing countries, where the health care provider-to-population ratio is very small^61^ and where community-level actors such as AFDs can play great roles.

It is not possible to solve nutritional problems in a sustainable way through supplementation in the form of capsules or tablets or nutritional therapeutic interventions. It would be too difficult to organize, and the costs would be too high; therefore, such a model-based NBCC intervention would lead to sustainable improvements in the nutritional status and overall health of mothers and their fetuses. Community health workers (CHWs) or AFDs are critical for improving access to different health services, as this study revealed that they can contribute significantly to NBCC interventions and health policy makers. Government and nongovernmental organizations need to continue actively involving them in organized ways with periodic performance measurements and improvements in their knowledge and skills.

There are several possible explanations for the significant improvement in nutritional status and GWG resulting from the NBCC intervention in this study; these include the use of HBM, husbands’ participation in education sessions, the use of the GALIDRAA counseling technique, teaching aids such as leaflets, cooking demonstration techniques and providing trimester-based nutrition education.

Once a pregnancy occurs in adolescence, it is desirable to successfully manage it by proper nutrition and all ANC services. Further studies are needed to explore what other behavioral change communication models can be used. These findings have wider practical implications for the need to implement adolescent nutrition guidelines to prevent intergenerational cycles of malnutrition that occur during adolescent pregnancy^66^. The results also imply the great potential of leveraging existing community-level structures, such as AFDs, for improving adolescent nutrition during pregnancy.

The strengths of this study include the use of community-based theory-based NBCC, cooking demonstrations, and the involvement of pregnant adolescents in the NBCC; however, the limitations includes, except for those concerning the measurement of MUAC, others were based on the pregnant adolescents’ recall, self-reports, and honesty in answering the questions.

## Conclusion

This study demonstrated that HBM-based NBCC combined with husband involvement through AFDs was effective at improving the nutritional status and gestational weight gain of pregnant adolescents. Utilizing AFDs ensures that the NBCC is a sustainable and low-cost intervention. Thus, it is recommended that the HBM be included in nutritional counseling guidelines. This reproducible NBCC intervention may be scaled up and sustained at a low cost through existing health systems and community structures.

## Data Availability

The datasets used and/or analyzed during the current study are available from the corresponding author upon reasonable request.

## Abbreviations

AFD: Alliance for development
AIC: Akaike information criterion
BCC: Behavior change communication
DID: Difference in difference
FAO: Food and Agricultural Organization
GWG: Gestational weight gain
HBM: Health belief model
HFIAS: Household Food Insecurity Access Scale
ICC: Intracluster correlation
IEC: Information Education Communication
LMIC: Low- and middle-income
MUAC: Mid-upper arm circumference
NBCC: Nutritional Behavior Change Communication
PCA: Principal component analysis
PHCU: Primary healthcare unit
SRS: Simple random sample
WDA: Women development army
WHO: World Health Organization
